# Undetected Neuromuscular Disease in Patients after Heart Transplantation

**DOI:** 10.3390/ijms25147819

**Published:** 2024-07-17

**Authors:** Biniam Melese Bekele, Elisabetta Gazzerro, Felix Schoenrath, Volkmar Falk, Simone Rost, Selina Hoerning, Yvonne Jelting, Ann-Kathrin Zaum, Simone Spuler, Jan Knierim

**Affiliations:** 1 Muscle Research Unit, ECRC Experimental and Clinical Research Center, Charité—Universitätsmedizin Berlin, corporate member of Freie Universität Berlin and Humboldt-Universität zu Berlin, Lindenberger Weg 80, 13125 Berlin, Germany; biniam.bekele@dhzc-charite.de (B.M.B.); elisabetta.gazzerro@charite.de (E.G.); 2 Max-Delbrück-Center for Molecular Medicine in the Helmholtz Association (MDC), 13125 Berlin, Germany; 3 Charité—Universitätsmedizin Berlin, corporate member of Freie Universität Berlin and Humboldt-Universität zu Berlin, Charitéplatz 1, 10117 Berlin, Germanyknierim@paulinenkrankenhaus.de (J.K.); 4 Deutsches Herzzentrum der Charité—Medical Heart Center of Charité and German Heart Institute Berlin, Department of Cardiothoracic and Vascular Surgery, Augustenburger Platz 1, 13353 Berlin, Germany; 5 DZHK (German Centre for Cardiovascular Research), Partner site Berlin, 13125 Berlin, Germany; 6 Translational Cardiovascular Technologies, Department of Health Sciences and Technology, Swiss Federal Institute of Technology (ETH), 8093 Zurich, Switzerland; 7 Institute for Human Genetics, University of Würzburg, 97074 Würzburg, Germany; 8 Berlin Institute of Health at Charité—Universitätsmedizin Berlin, Charitéplatz 1, 10117 Berlin, Germany; 9 Sana Paulinenkrankenhaus, Department of Internal Medicine and Cardiology, Dickensweg 25-39, 14055 Berlin, Germany

**Keywords:** heart transplantation, skeletal muscle weakness, cardiomyopathy, nexilin, myosin heavy chain 7, titin

## Abstract

(1) Heart transplantation (HTX) improves the overall survival and functional status of end-stage heart failure patients with cardiomyopathies (CMPs). The majority of CMPs have genetic causes, and the overlap between CMPs and inherited myopathies is well documented. However, the long-term outcome in skeletal muscle function and possibility of an undiagnosed underlying genetic cause of both a cardiac and skeletal pathology remain unknown. (2) Thirty-nine patients were assessed using open and standardized interviews on muscle function, a quality-of-life (EuroQol EQ-5D-3L) questionnaire, and a physical examination (Medical Research Council Muscle scale). Whole-exome sequencing was completed in three stages for those with skeletal muscle weakness. (3) Seven patients (17.9%) reported new-onset muscle weakness and motor limitations. Objective muscle weakness in the upper and lower extremities was seen in four patients. In three of them, exome sequencing revealed pathogenic/likely pathogenic variants in the genes encoding nexilin, myosin heavy chain, titin, and SPG7. (4) Our findings support a positive long-term outcome of skeletal muscle function in HTX patients. However, 10% of patients showed clinical signs of myopathy due to a possible genetic cause. The integration of genetic testing and standardized neurological assessment of motor function during the peri-HTX period should be considered.

## 1. Introduction

Heart transplantation (HTX) is the gold standard for patients with end-stage heart failure. Compared to conservative treatment, HTX considerably improves cardiac function, quality of life, and overall survival [1]. Indications for HTX include ischemic heart disease, congenital cardiac defects, heart valve abnormalities, and, most frequently, cardiomyopathy (CMP) [2]. CMP is an overarching term for a diverse group of diseases characterized by structural and functional abnormalities of the heart muscle in the absence of secondary causes that are sufficient to cause the observed myocardial abnormality [3,4]. Despite the significant amount of acquired cardiomyopathies, genetic causes account for a large proportion of CMP patients. Advances in genetic diagnostic techniques have enabled the characterization of these causes. Over the last three decades, research has primarily focused on the identification of monogenetic Mendelian disease genes, often with an autosomal dominant pattern of inheritance. However, other inheritance patterns, including autosomal-recessive, X-linked, and mitochondrial patterns, have also been observed. Furthermore, significant genetic and allelic heterogeneity has been observed with incomplete and age-related penetrance [5,6].

CMP is phenotypically classified into the following subgroups: hypertrophic cardiomyopathy (HCM), dilated cardiomyopathy (DCM), restrictive cardiomyopathy (RCM), arrhythmogenic right ventricular cardiomyopathy (ARVC), cardiomyopathy with other extra-cardiac manifestations, and left ventricular noncompaction (LVNC) [7]. HCM is among the most extensively studied cardiomyopathies, with over a thousand mutations identified in various genes. These mutations usually follow an autosomal dominant inheritance pattern, exhibiting significant variation in expression and penetrance [8,9]. Around 40% of DCMs have a genetic basis with a heterogenous group of genes encoding proteins involved in different functions, including sarcomere integrity and cytoskeletal architecture [7,10]. The most common are pathologic variants in *Titin (TTN)*, *Lamin A/C (LMNA,)* and *beta-myosin heavy chain (MYH7)* [9]. Familial LVNC is characterized by mutations in sarcomere-encoding genes, with mutations in *MYH7*, *TTN*, and *myosin-binding protein C* (*MYBPC3*) being the most prevalent [9]. Several mutations in genes encoding desmosomale proteins have been implicated in ARVC, whereas RCM is relatively rare with significant genetic overlaps with other CMPs, particularly HCM [9].

Muscular dystrophies (MDs) are a related but different group of genetic disorders. The vast majority are caused by autosomal-recessive genetic traits and characterized by the progressive wasting of skeletal muscle. Affected people frequently have severe motor limitations, such as an inability to walk and difficulty breathing and swallowing, which significantly reduce their life expectancy. The therapeutic landscape for MDs has been changing rapidly in the last decade. A few gene replacement therapies have been approved, paving the way for adequate treatment of MD patients. Microdystrophin gene replacement therapy was approved by the Federal and Drug Administration in June 2023 for Duchenne muscular dystrophy [11]; gene replacement and gene editing therapies are being tested in clinical trials for limb–girdle muscular dystrophies (http://www.clinicaltrials.gov: NCT05588401 NCT05230459, NCT05906251, NCT05876780, and NCT05876780; accessed on 1 July 2024). Different pharmacological strategies (givinostat, a blocker of HDAC acetylation, ribitol, and steroids) have been approved or are in the last phases of clinical testing (http://www.clinicaltrials.gov: NCT0178350 NCT00527228 NCT03373968, and NCT05933057; accessed on 1 July 2024).

The overlap between CMPs and MDs has long been recognized. Mutations in the *dystrophin* gene, *DMD*, cause the progressive loss of skeletal muscle strength and cardiac function [12]. *LMNA* gene mutations have been shown to cause skeletal myopathy and cardiac disease [13,14]. *Desmin (DES)* mutations cause atypical cardiomyopathy and skeletal myopathy [15]. A novel missense mutation in *αB-crystallin (CRYAB)* causes restrictive cardiomyopathy in combination with skeletal myopathy [16]. In clinical practice, an underlying myopathy can be a relative contraindication and a challenge to HTX; however, since the clinical onset of the skeletal and heart diseases can be very asynchronous, a lack of accurate genetic testing can result in a missed diagnosis.

We were confronted with two patients who had undergone HTXs more than ten years prior and had recovered well. Approximately six years after the HTXs, they noticed new-onset motor difficulties. One patient became wheelchair-bound three years later; the other became wheelchair-bound after five years. In both patients, a mutation in *Titin* (TTN, *188840), a gene encoding the giant protein titin, was subsequently identified. TTN mutations might well be responsible for cardiac and skeletal muscle failure. Their complete dependence on external help a few years after the HTXs could not have been foreseen without appropriate genetic testing.

We asked ourselves whether this new onset of skeletal muscle wasting due to genetic causes many years after these HTXs was an isolated incidence. In a collaborative effort, the prospective study, MuSCor, systematically assessed 39 patients who underwent HTXs between five and fifteen years prior for subjective complaints and objective signs and performed whole-exome DNA sequencing when muscle weakness was evident.

## 2. Results

### 2.1. Patient Characteristics

Thirty-nine patients were included in this study, all of whom underwent orthotopic HTXs between 2000 and 2015. The median age at HTX was 32.5 (19.5–46) years, and 79% of the patients were male. The most common indication for HTX was DCMP. None of the patients reported a family history of neuromuscular disease, while 13% had a family history of cardiac disease. Two patients had undergone a second HTX. In one of these two patients, the second procedure was a combined heart and kidney transplantation (see Table 1).

At the time of their inclusion in this study, all the patients were free of heart failure symptoms. A mean duration of 14.5 (11–17) years had passed since their HTXs. The majority of patients had a preserved left ventricular ejection fraction (LVEF) (92.5%) and no significant valvular dysfunction (87.5%). Most of the patients were taking statins (85%) and steroids (57.5%), and all of the patients took immunosuppressants (Table 2).

### 2.2. Quality of Life

**Figure 1:**
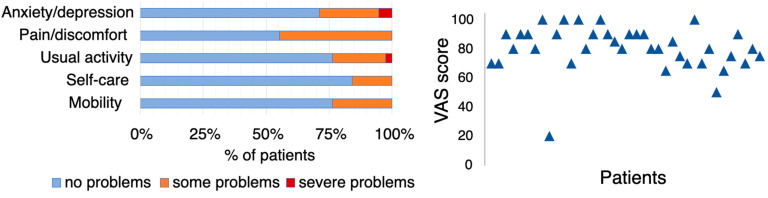
EQ-5D-3L questionnaire results: Scores on the five dimensions (**left**). Scores from the Visual Analogue Scale (VAS) (**right**).

In the EQ-5D-3L questionnaire, 23.7% of the patients reported problems/difficulties (defined as either moderate or severe) with mobility, 15.8% with self-care, and 23.7% with their usual activities. A total of 44.7% reported pain/discomfort, and 28.9% reported anxiety/depression. In the EQ-VAS, the patients rated their overall health at an average score of 80.4 ± 15.3 (Figure 1).

### 2.3. Motor Function and Genetic Results

At the time of their inclusion in this study, 15% of the patients reported experiencing skeletal muscle weakness, while 17.9% reported perceiving reduced muscle strength compared to their peers. Regarding their musculoskeletal functions in daily life, 15% reported difficulties climbing stairs, 12.5% reported fine motor limitations, such as difficulties holding a pen or opening bottles, and 10% reported difficulties getting up from a seated position. For details, see Table 3.

**Table 3:** Limitations in musculoskeletal functions in daily life at the patients’ time of inclusion in this study.

Muscle Complaints	*n* (%)
Muscle weakness	6 (15.4%)
Reduced muscle strength compared to peers	7 (17.9%)
Difficulty raising arms above the head	2 (5%)
Difficulty getting up from sitting	4 (10.3%)
Difficulty standing/walking on toes	3 (7.5%)
Difficulty climbing stairs	6 (15.4%)
Difficulty in fine motor activities	5 (12.8%)
Difficulty swallowing food	0
Difficulty breathing	2 (5%)
Difficulty supporting head	0
Family history of musculoskeletal disease	0
Stiffness	2 (5%)
Tingling sensation in the extremities	2 (5%)
Numbness in the extremities	1 (2.6%)

During the neurological motor examination and assessment of muscle strength using the MRC scale, four patients demonstrated objective muscle weakness. A summary of these patients is provided in Table 4.

- Patient 1 (#012)

This 30-year-old female patient initially underwent HTX when she was 1 year old for DCMP followed by a combined kidney transplantation and re-HTX when she was 14 for cardiac allograft vasculopathy and chronic kidney disease. The patient had no family history of musculoskeletal complaints. After a period of regained exercise tolerance, new musculoskeletal symptoms appeared when she was 24, namely difficulties climbing stairs, lifting her arms above her head, and opening bottles, and she had a maximum unaided walking distance of 1 km. The physical examination revealed muscle weakness, which was more pronounced in the proximal leg muscles, with impaired knee bending and heel walking (MRC symmetrical 3+/5 for knee flexors/plantar extensors, symmetrical 4/5 in hip flexors/hip extensors/plantar flexors, and symmetrical 4+/5 in toe extensors/flexors). No muscle atrophy or abnormal muscle activity was observed. At the time, the patient had terminal chronic kidney disease and was listed for kidney re-transplantation.

**Figure 2:**
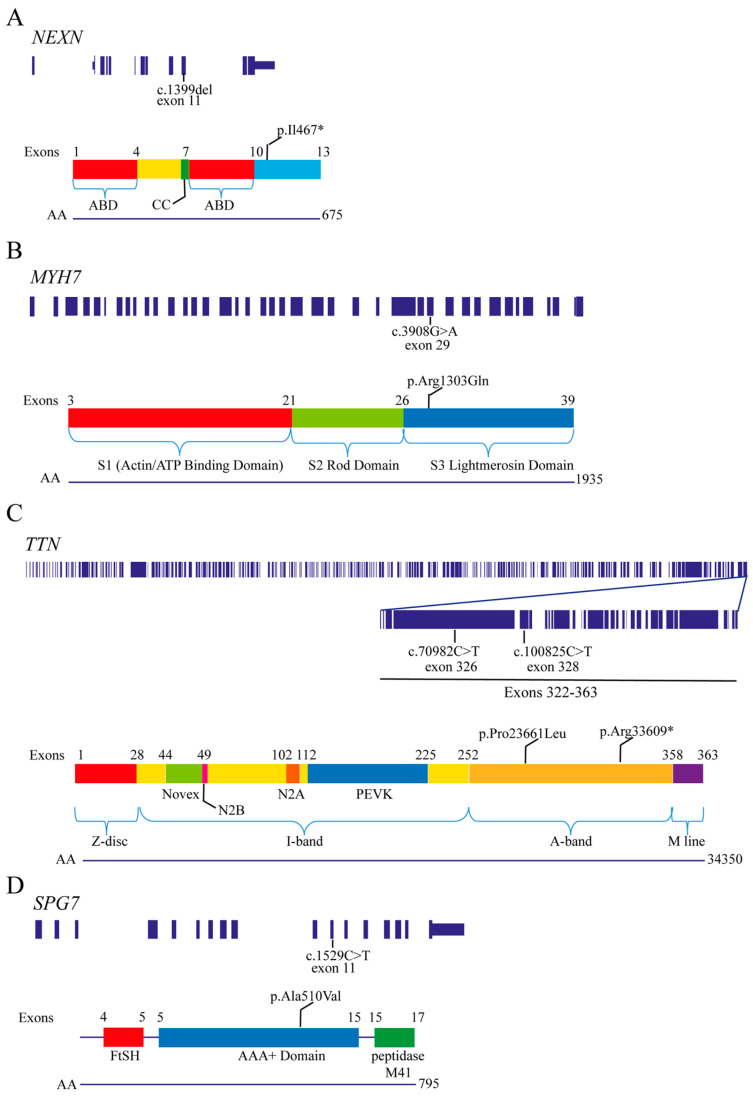
Schematic representation of exons and protein domains with localization of the likely pathogenic variants. (**A**) Human *NEXN* transcript, NM_ 144573. For protein domains, data were derived from the rat protein sequence [26]. Rat and human *NEXN* sequences are 86% identical (Ensembl). (**B**) Human *MYH7* transcript, NM_000ß257. (**C**) Human *TTN* transcript, NM_001267550. Only exons are depicted. (**D**) Human *SPG7* transcript, NM_ 003119. Modified from the UCSC Genome Browser (www.genome.ucsc.edu) and the literature [27,28,29,30]. AA—amino acids; ABD—F-actin binding domain; CC—coiled-coil region; *NEXN*—nexilin; *TTN*—titin; *MYH7*—myosin heavy chain 7; *SPG7*—spastic paraplegia 7. *—Stop codon.

**Figure 3:**
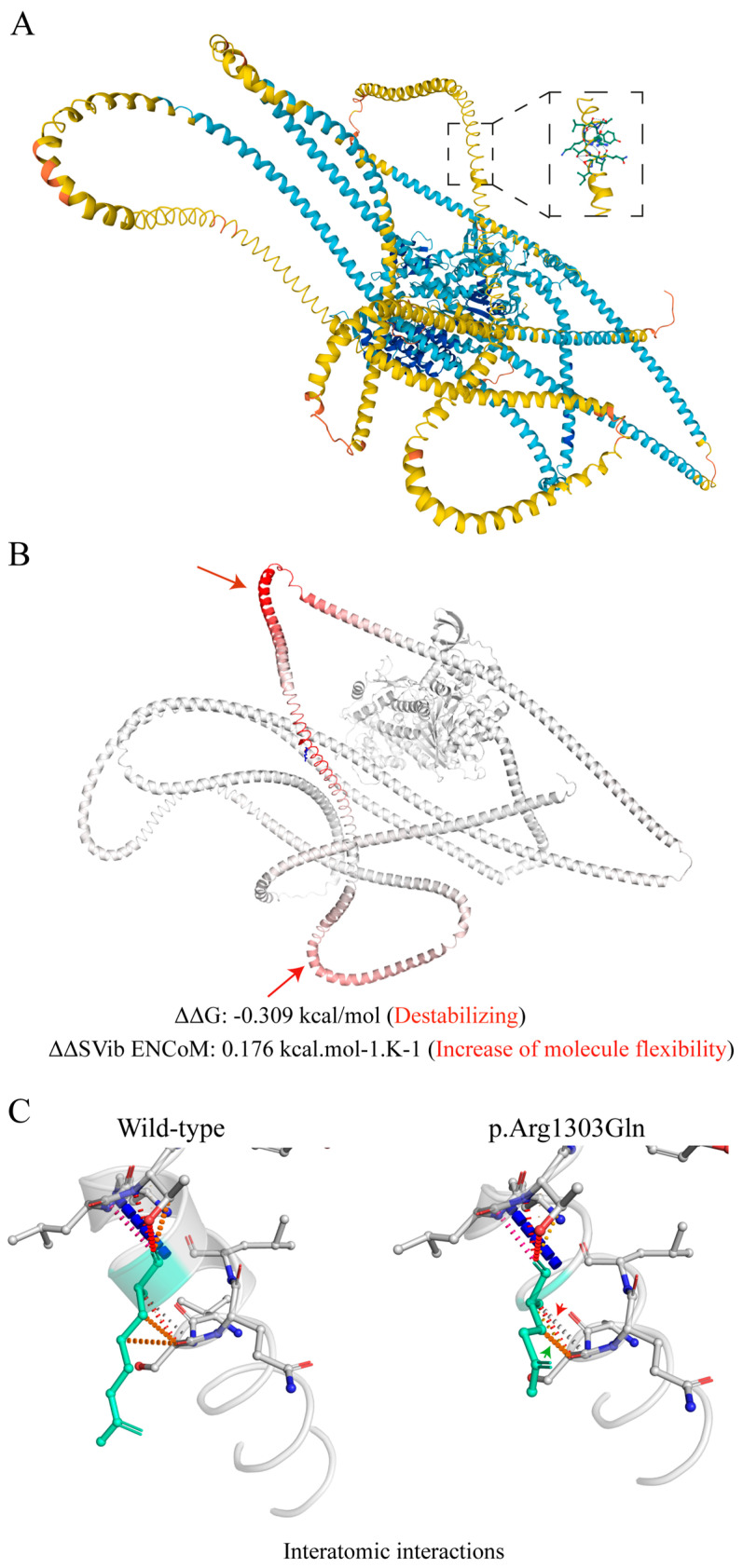
Prediction analysis of the impact of *MYH7*c.3908G>A, p.(Arg1303Gln) on protein dynamics and stability resulting from vibrational entropy changes. (**A**) Predicted alpha-fold myosin heavy chain 7 protein structure (Uniprot) [31]. The inset shows the amino acid arginine at position 1303. (**B**) Prediction outcome on entropy energy change (ΔG-kcal/mol) and vibrational entropy change (ΔΔSVib ENCoM-kcal/mol^−1^/K^−1^) between wild-type and p.(Arg1303Gln) mutant protein. Arrows indicate amino acid sequences characterized by increased flexibility. (**C**) Changes of interatomic interactions. Green arrowhead indicates loss of one weak hydrogen bond, and red arrowhead indicates increase in ionic interactions (Dynamut [17]).

Exome sequencing revealed two pathogenic variants: *NEXN* c.1399del (NM_144573.3, *613121; ACMG score: PVS1, PM2) and *MYH7* c.3908G>A (NM_000257.3, 160760) (Table 5). *NEXN* encodes nexilin, an actin-binding filament whose heterozygous deletion c.1399del results in a premature stop codon at amino acid (aa) position 467 (Figure 2A). In *MYH7,* encoding myosin heavy chain 7 beta, the heterozygous missense variant c.3908G>A (ACMG score: PM2, PM1, PP3, PP5) results in the aa exchange p.(Arg1303Gln) in the C-terminal light merosin domain (Figure 2B). The affected base and aa are highly conserved across eleven species (Ensembl) (Table 5). A prediction analysis using the Dynamut algorithm indicated that the *MYH7* p.(Arg1303GLN) mutant exhibits a destabilizing effect with a negative change in protein entropy (ΔΔG: −0.309 kcal/mol) and increased flexibility (ΔΔSVib ENCoM: 0.176 kcal.mol^−1^·K^−1^), both related to changes in interatomic hydrogen bonds and ionic interactions (Figure 3A–C) [17]. According to the three-dimensional structure prediction model AlphaFold (https://alphafold.ebi.ac.uk: accessed on 1 July 2024), Arginine 1303 is located in an unresolved loop (pLLDT = 62.5), and the alpha pathogenicity score of the Arg1303Gln variant corresponds to 0.548, thus falling in the range of uncertain significance. A stretch of 20 amino acid residues, including the wild-type or the p.Arg1303Gln mutant at the midpoint, was analyzed via the Deepcoil neural network. The probabilities of a coiled-coil structure corresponded to 0.917% and 0.905%, respectively, in the wild-type and mutant protein.

- Patient 2 (#034)

This 72-year-old male patient underwent HTX at age 52 due to DCMP and developed musculoskeletal symptoms at age 68. He described feeling fatigued, having pain in the proximal and distal leg muscles after light straining, back muscle stiffness, and difficulty climbing stairs. His walking distance was reduced to 300 m. A physical examination revealed difficulty in performing the knee-bending test, a positive Gower’s sign, atrophy of the temporalis muscles, weakness of the facial muscles, and a high-arched palate (MRC symmetrical 4+/5 in shoulder elevators/knee flexors/knee extensors and 4/5 in hip flexors/hip extensors).

The first-step screening of exome sequencing showed a nonsense and missense variant in the *TTN* gene (NM_001267550.2), encoding the cardiac and skeletal muscle protein titin. Both are heterozygous and rarely present in healthy populations. The nonsense variant c.100825C>T results in the premature stop p.(Arg33609*), probably indicating NMD or protein truncation, and is listed as likely pathogenic and pathogenic. The missense variant c.70982C>T p.(Pro23661Leu) was located in exon 327 of the A-band region (Figure 2B). The CNV analysis did not yield any information of interest. In the core cardiomyopathy panel, we also identified the missense VUS c.278T>C in the dominant *TNNI3* gene, which encodes for the cardiac isoform of troponin I (transcript levels are 720-fold higher in cardiac than in skeletal muscle). Pathogenic variants in TNNI3 (* 191044) are described in patients affected by hypertrophic cardiomyopathy [18]. The in silico programs predicted a possibly damaging effect of the variant, which was reported in ClinVar with uncertain significance (Supplementary Table S1). In the second step of the analysis, a missense VUS in the dominant *FBN2* gene, encoding the fibrillin-2 protein, was detected. *FBN2* loss-of-function causes musculoskeletal features of Marfan syndrome [19]. However, *FBN2* has a very low expression in cardiac muscle tissue. Similarly, a possibly damaging missense variant was identified in the gene *HADHA*, encoding the hydroxyacyl-CoA dehydrogenase, whose recessive gene defect causes pediatric long-chain 3-hydroxyacyl-CoA dehydrogenase deficiency [20].

- Patient 3 (#009)

This 58-year-old male patient underwent HTX at age 47 due to DCMP and began experiencing muscle weakness at age 57. He described difficulties rising from a seated position and climbing stairs, frequent stumbling accidents, and an unaided walking distance limited to only 200 m. He had no recollection of similar complaints in any other family members. Upon physical examination, he showed the following MRC scale results: symmetrical grade 4/5 for proximal and distal muscles of the upper limb, symmetrical grade 3+/5 in hip flexors and hip extensors, 4−/5 in knee flexors and extensors on the left side, and 3+/5 in the same muscle groups on the right side. In addition, atrophy of the quadriceps femoris muscle, more pronounced on the right side, was present. Deep tendon reflexes were reduced but present.

In the first-step analysis, no pathogenic variants were identified except a VUS in the POMK (* 615247) gene, which encodes the protein O-mannose kinase, a protein required for proper glycosylation and function of the dystroglycan complex on the muscle membrane. Autosomal-recessive POMK-related dystroglycanopathy is characterized by a wide range of clinical presentations from congenital muscular dystrophy to adult-onset limb girdle muscular dystrophy [21]. Major heart involvement as well as disease onset in heterozygous carriers has not yet been described. The HPO search found a probably damaging variant in *LGL4*, syntaxin-binding protein 5, which is associated with a neurodegenerative phenotype [22]. Another interesting finding is the variant c.56C>G p.(Ser19Trp), which had a missense change in the *GATA5* gene; GATA-binding protein loss-of-function mutations in this gene are associated with congenital heart defects [23].

Based on the preliminary inconclusive genetic results and the pronounced atrophy of the patient’s quadriceps, a muscle biopsy is a crucial next step and could find evidence of an acquired inflammatory/degenerative myopathy, such as inclusion body myositis, the most common sporadic muscle disorder in the elderly [24].

- Patient 4 (#028)

This 70-year-old male patient underwent HTX at age 60 due to DCMP and began experiencing new musculoskeletal symptoms at age 69. He reported leg muscle weakness and pain, difficulty climbing stairs, impairment in fine motor activities such as opening bottles, stiffness in his fingers, and a walking distance limited to 500 m. He had no recollection of similar complaints or muscle diseases in the family. The physical examination revealed an impaired tiptoe and heel walking test, knee bending test, and a positive Gower’s sign. Manual muscle testing revealed 4+/5 on the right side and 5−/5 in the left hip flexors and hip extensors, as well as 4+/5 in the knee flexors and knee extensors bilaterally.

Even though no likely pathogenic mutations were identified in the first ROI analysis, the second analysis using HPO terms revealed a pathogenic mutation in the recessive *SPG7* gene (NM_003119) encoding the mitochondrial protein paraplegin. This c.1529C>T p.(Ala510Val) missense variant has already been described in the homozygous or compound heterozygous state in patients affected by adult-onset cerebellar ataxia [25] (Figure 2D). Even though paraplegin is expressed in heart tissue, its possible causative role in cardiomyopathies has not been described yet.

**Table 5:** Pathogenic or likely pathogenic variants in patients 012, 028, and 034.

	Gene OMIM^®^	Transcript	Variant	Allele Frequency % (gnomAD) [32]	In Silico Prediction		Database		Conservation (Ensembl) [33]	
					SIFT [34]	Mutation Taster [35]	Polyphen-2 [36]	ClinVar [37]	HGMD [38]	
Patient 1 (012)	MYH7 *160760	NM_000257.3	c.3908G>A p.(Arg1303Gln)	0.0004	del.	disease causing	probably damaging	Uncertain	(pathogenic aa exchange at same position) [39]	highly (nt + aa)
	NEXN *613121	NM_144573.3	c.1399del p.(Ile467*)	0.0032	probably premature stop with NMD		NA	NA	NA	
Patient 2 (034)	TTN *188840	NM_001267550.2	c.100825C>T p.(Arg33609*)	0.0004	premature stop probably indicating NMD or protein truncation		Likely pathogenic	uncertain (DCMP)	NA	
			c.70982C>T p.(Pro23661Leu)	0.00041	NA	NA	probably damaging	uncertain	NA	highly (nt)
Patient 4 (028)	SPG7 *602783	NM_003119	c.1529 C>T p.(Ala510Val)	0.2899	del.	NA	probably damaging	Pathogenic; likely pathogenic; uncertain	NA	highly

MYH7—myosin heavy chain 7; NEXN—nexilin; SPG7—spastic paraplegia 7; NA—not available; AA—amino acid; NMD—nonsense-mediated decay; nt—nucleotide(s); DCMP—dilated cardiomyopathy; del—deleterious.

## 3. Discussion

The MuSCor study evaluated the skeletal muscle function of 39 cardiomyopathy patients 5 to 15 years after HTX. A total of 17.9% of the patients reported subjective motor function limitations following an initial period of regained exercise tolerance. In four (10.5%) patients, we confirmed clinical signs of impaired muscle strength in the upper and lower extremities, and in three (7.7%) patients, we identified likely pathogenic/pathogenic variants in the *NEXN*, *MYH7*, *TTN*, and *SPG7* genes.

Skeletal muscle weakness after HTX can be caused by a variety of factors, including preexisting structural and functional changes related to chronic heart failure and deconditioning due to inactivity. The long-term use of drugs like immunosuppressants and statins has also been shown to be myotoxic [40,41,42]. However, research on long-term skeletal muscle function after HTX is limited. Some studies have found that changes such as a lower proportion of type I muscle fibers persist over time [43,44]. Fernandes et al. [45] reported a gradual increase in respiratory muscle strength, hand grip, and muscle mass in a prospective study that followed up 23 patients for 1.5 to 3 years after HTX. A recent study by Regamey et al. [46] followed up 48 patients for nine years after HTX and reported that, while 20% of the patients remained in the sarcopenic range, all showed a steady increase in appendicular lean mass and exercise tolerance. Our findings are consistent with these studies, in which only 17.9% of participants exhibited motor function limitations in their daily life.

Myopathies are a relative contraindication to HTX and present a significant challenge for heart transplant teams and neurologists during the HTX evaluation process. A reduced life expectancy, an increased operative risk due to respiratory muscle impairment, a prolonged need for ventilatory support, a higher rate of postoperative complications, potential graft involvement due to underlying myopathy, and an inability to participate in rehabilitation can all severely compromise successful HTX [47]. A case-by-case evaluation is more often required to avoid life-threatening complications [48]. Therefore, the identification of asymptomatic or paucisymptomatic inherited skeletal muscle diseases and genetic diagnosis in end-stage cardiomyopathies is crucial in the pre-HTX workflow. A thorough diagnostic process whose findings are communicated effectively enables patients to give their fully informed consent and understand their quality-of-life expectations following HTX.

Our study identified three patients suffering from a neuromuscular disorder whose symptoms developed years after their HTXs. None of the patients had a prior genetic diagnostic workup. The *NEXN* c.1399del results in a premature stop codon and loss of nexilin, a protein highly expressed in the human heart and skeletal muscle and localized at the sarcomeric Z-disc [26,49]. Several heterozygous mutations in *NEXN* were described in DCMP and HCMP [49,50,51,52,53]. Z-disks in nexilin-deficient skeletal muscle cells are destabilized in a workload-dependent manner, highlighting the unique role of nexilin in protection from mechanical trauma [52]. However, a skeletal muscle disease phenotype has not yet been described. It is conceivable that the severe cardiac phenotype masks skeletal muscle findings in NEXN knockout mice, showing a drastically reduced life span due to DCMP [54]. Mutations in *MYH7* are known to cause DCMP and skeletal muscle dystrophy [55,56]. The variant *MYH7* c.3908G>C, p. (Arg1303Pro) was reported previously in a patient suffering from left ventricular non-compaction cardiomyopathy [39] and is predicted to have a destabilizing effect on protein structure. However, whether this destabilization leads to functional loss cannot be predicted. Furthermore, it is not possible to determine precisely which gene mutation caused the phenotype in our patient; however, combined digenic variants of *MYH7* and *NEXN* have already been described [57].

Titin is the largest protein in the human body. Heterozygous mutations that truncate full-length titin are the most common genetic cause of severe and familial DCMP, accounting for approximately 25% of cases [58], and c.100825C>T had been, in fact, already identified in one patient affected by DCMP [59]. In addition to cardiomyopathies, mutations in the *TTN* gene also cause muscular phenotypes, and the two conditions may coexist. We also found several VUSs in all four patients, including variants in genes whose loss of function is a known mechanism of cardiomyopathies, such as *ANKRD1* and *TNNI3*, but whose role in skeletal muscle has not yet been fully described. We also discovered missense changes in *RYR3*, a gene that is highly expressed in skeletal muscle but has not yet been linked to a genetic muscle disease. However, these sequence variations did not meet the ACMG criteria for pathogenicity (Supplementary Table S1).

Patient #4 carried the pathogenetic variant c. 1529C>T in the SPG7 gene in a heterozygous state. SPG7 encodes the mitochondrial protein paraplegin, a component of the m-AAA protease. In homozygosity or compound heterozygosity, c.1529C>T causes adult cerebellar ataxia. The heterozygous state was described in three patients affected by the I and II motoneuron disorder amyotrophic lateral sclerosis [25]. Hypertrophic and dilated cardiomyopathy are clinical manifestations of a mitochondrial genetic disorder. Even though paraplegin is expressed in heart tissue, its possible causative role in cardiomyopathies has not been described yet.

Our study has a few limitations. The sample size was small, and genetic testing was performed only in patients with subjective and objective muscle weakness. The presence of similar mutations in other asymptomatic patients is unknown. Our study did not include further analyses, such as a linkage analysis or protein expression changes. In patients with clinical evidence of skeletal muscle disease, a histological muscle analysis would help in the diagnosis of inherited and acquired disorders. Moreover, due to the small sample size, out study did not include a sub-group analysis based on confounding factors, such as the use of myotoxic agents or donor and recipient clinical factors. Thus, interpretations of our findings should consider these factors.

New-onset muscle weakness in CMP patients following HTX can be multifactorial. Our study highlights one possible explanation given the well-established genetic causes of CMP and the overlap between CMP and MDs. However, other acquired etiologies including but not limited to inflammatory causes, drug-induced changes, age/mobility-related degeneration, underlying chronic disease changes, or a combination of factors are also a possibility. Therefore, a comprehensive clinical evaluation and work-up are crucial to determine the exact etiology.

## 4. Materials and Methods

### 4.1. Patients and Data Collection

**Figure 4:**
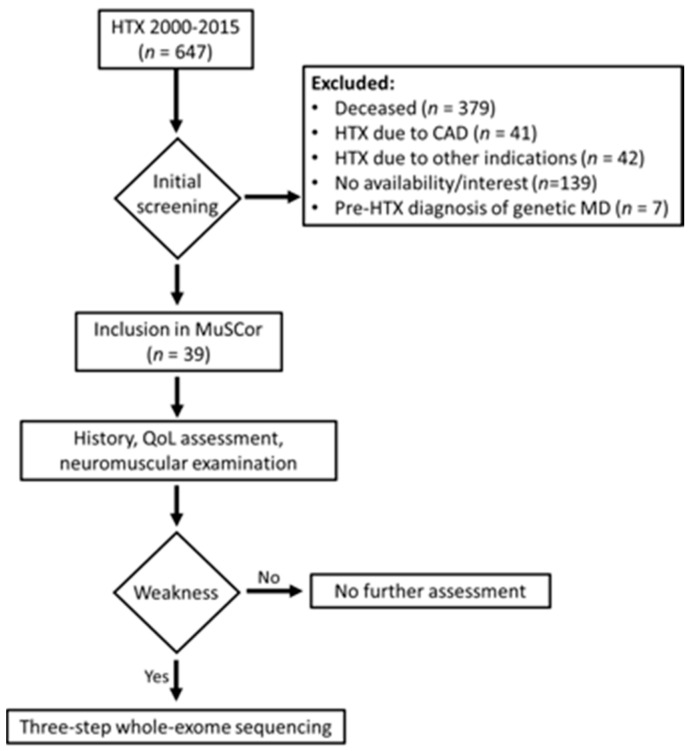
MuSCor design. HTX—heart transplantation; CAD—coronary artery disease; MD—muscular dystrophy; QoL—quality of life.

Patients had to meet the following inclusion criteria to be eligible for the MuSCor prospective observational study: having had heart transplantation between the years 2000 and 2015, being older than 18 years old, having had no prior diagnosis of hereditary muscle disease, and being able to provide signed informed consent. The study was approved by the institutional ethical review board (EA1/104/21). Out of a total of 647 patients eligible for initial screening, 39 patients were included (see Figure 4).

Medical records including demographic data, co-morbidities, and therapy at the pateints’ time of inclusion in the study were reviewed. Quality of life was assessed using the self-reported German version of the EuroQol EQ-5D-3L [60] questionnaire, which comprises a descriptive system and a visual analogue scale. Creatine kinase (CK) levels were measured in all patients and reported as µkat/L.

### 4.2. Motor Function

Their medical history on their motor function during activities in their daily life was collected. A neuromuscular assessment was performed using standard physical examination techniques and the modified Medical Research Council (MRC) scale [61]. The MRC is commonly used in clinical practice and examines 28 movements of the neck, trunk, and upper and lower limbs and scores muscle strength from grade 5 (normal strength) to grade 0 (no visible contraction).

### 4.3. Genetic Analysis

Whole-exome sequencing was performed using Illumina^®^ DNA Prep with Enrichment (Illumina, Inc. San Diego, CA, USA) and xGen Exome Research Panel (Integrated DNA Technologies, Coralville, IA, USA) according to the manufacturers’ instructions. The patients’ exomes were screened for potentially disease-causing variants in a three-stage analysis. First, a region of interest (ROI) was defined to include a specific set of genes already associated with cardiac and muscular disease (core cardiomyopathy: 92 genes, core myopathy: 131 genes) (Supplementary Tables S2 and S3). The ROI search had a twenty-fold coverage in >99% of reads. In a second step, two HPO (Human Phenotype Ontology) terms “Cardiomyopathy” (HP:0001638) and “Abnormality of the musculature of the limbs” (HP:0009127) were used (Supplementary Tables S4 and S5). This search had a twenty-fold coverage of >95%. In the third step, exomes were scanned for copy number variations using Gensearch^®^NGS. The variants were evaluated in accordance with the American College Medical Guidelines (ACMG) [62].

Furthermore, several tools including SIFT (http://www.sift.jcvi.org/: accessed 1 April 2021) [34], Mutation Taster (www.mutationtaster.org: accessed 1 March 2021) [35], Polyphen-2 (www.genetics.bwh.harvard.ed/pph2: accessed 1 April 2021) [36], Genome Aggregation Database (gnomAD) (http://gnomad.broadinstitute.org: accessed 1 April 2021) [63], ClinVar (www.ncbi.nlm.nih.gov/clinvar: accessed 1 April 2021) [37], Human Gene Mutation Database (HGMD, www.hgmd.cf.ac.uk: accessed 1 April 2021) [38], Online Mendelian Inheritance in Man compendium (OMIM, www.omim.org: accessed 1 April 2023), Expression Atlas from the European Informatic Institute (EMBL_EBI, www.ebi.ac.uk: accessed 1 April 2021) [64], and ENSEMBl genome browser (www.ensembl.org: accessed 1 April 2023) [33] were used. The Dynamut software (www.biosig.unimelb.edu.au/dynamut/prediction: accessed 1 April 2024) was used to study and represent the impact of the likely pathogenic variants on protein stability, flexibility, and conformation [17]. In addition, Alphafold (https://alphafold.ebi.ac.uk: accessed on 1 July 2024), a 3D structure prediction model, and DeepCoil (https://toolkit.tuebingen.mpg.de/tools/deepcoil: accessed on 1 July 2024), a neural network method for prediction of coiled coils based on sequence, were used. Likely pathogenic and VUSs (variants of unknown significance) in dominant inherited genes as well as single-allele variants in recessively inherited genes were considered and are shown in Table 5. Only VUSs for which multiple computational analysis supported a deleterious effect are further described in the results section. For a detailed overview of the VUSs, see Supplementary Table S1.

### 4.4. Statistical Analysis

Demographic and clinical data are presented using descriptive statistics. For the EQ-5D-3L data, proportions of categorical responses for the five EQ-5D dimensions were reported. The visual analogue scores were summarized using descriptive statistics.

## 5. Conclusions

Our findings support the importance of genetic testing not only to diagnose underlying cardiac disease but also to determine the potential involvement of skeletal muscles. Further studies with a larger cohort of patients and in vitro/in vivo characterizations of each mutation and the associated functional changes are required. This information is crucial for patients and the whole clinical team in HTX evaluation and post-HTX care. Impairment in motor function significantly affects quality of life and hinders pateints in making the lifestyle modifications needed to optimize their cardiovascular risk factors following HTX. Thus, neurological examinations focused on motor function should become an integral part of the follow-up care for HTX patients.

## Figures and Tables

**Table 1:** Patient characteristics at the time of HTX (*n* = 39).

Characteristics	
Age at HTX (yrs.)	32.5 (19.5–46)
Sex	
Male	30 (79%)
Female	8 (21%)
Family history of cardiac disease	5 (13%)
Family history of neuromuscular disease	0
Pre-transplantation chronic illness	
Hypertension	27 (71%)
Type 2 diabetes mellitus	1 (2.6%)
Cerebrovascular disorders	2 (5.3%)
Indication for HTX	
Dilated cardiomyopathy	34 (89.5%)
Hypertrophic cardiomyopathy	1 (2.6%)
Arrhythmogenic cardiomyopathy	2 (5.3%)
Left ventricular noncompaction cardiomyopathy	1 (2.6%)

Data are presented as median (interquartile range) and *n* (%) for categorical variables. HTX—Heart transplantation.

**Table 2:** Patient characteristics at their time of inclusion in this study.

Characteristics	
Age at study inclusion (yrs.)	47 (35.8–58)
Years since HTX	14.5 (11–17)
BMI (kg/m^2^)	26.7 ± 4.5
BMI group	
Underweight (<18.5 kg/m^2^)	1 (2.5%)
Normal (18.5–24.9 kg/m^2^)	14 (35%)
Overweight (25–29.9 kg/m^2^)	13 (32.5%)
Obese (>30 kg/m^2^)	10 (25%)
LVEF %	
≥50%	37 (92.5%)
40–49%	1 (2.5%)
Significant valvular dysfunction	
None	35 (87.5%)
Tricuspid valve dysfunction	3 (7.5%)
Comorbidities	
Chronic kidney disease	23 (57.5%)
with dialysis	2 (5%)
Polyneuropathy	3 (7.5%)
Endocrine disorders	
Type 2 diabetes Mellitus	9 (22.5%)
Thyroid dysfunction	
Hypothyroidism	5 (12.5%)
Hyperthyroidism	1 (2.5%)
CK value (µkat/L)	1.5 (1.1–2.4)
Statins	
None	6 (15%)
Fluvastatin	10 (25%)
Atorvastatin	15 (37.5%)
Pravastatin	1 (2.5%)
Simvastatin	4 (10%)
Rosuvastatin	1 (2.5%)
Immunosuppressants	
Steroids	23 (57.5%)
Cyclosporin	22 (55%)
Tacrolimus	8 (20%)
Mycophenolate mofetil	24 (60%)
Everolimus	20 (50%)

Data are presented as mean ± standard deviation, median (interquartile range) for continuous variables, and *n* (%) for categorical variables. BMI—body mass index, LVEF—left ventricular ejection fraction, CK—creatine kinase, HTX—heart transplantation.

**Table 4:** Summary of demographics and physical findings of the four patients with muscle weakness.

**Study ID**	**Sex,** **Age** **(yrs.)**	**Age** **at HTX** **(yrs.)**	**HTX** **Indication**	**Onset of Muscle Symptoms** **after HTX (yrs.)**	**Pattern of Weakness**	**CK Levels** **(µkat/L)**	**Statins/** **Immunosuppressants**
012	♀, 30	14 *	DCMP	10	ULs: prox LLs: prox	0.85	Fluvastatin, MMF, Tac, prednisolone
034	♂, 72	52	DCMP	16	ULs: prox	1.83	Atorvastatin, Tac, MMF, prednisolone
009	♂, 58	47	DCMP	10	LLs: prox	5.48	Fluvastatin, CYA, everolimus and prednisolone
028	♂, 70	60	DCMP	9	LLs: prox	2.2	Everolimus, Tac

CK—creatine kinase; DCMP—dilated cardiomyopathy; HTX—heart transplantation; LLs—lower limbs; prox—proximal; ULs—upper limbs; yrs.—years; MMF—mycophenolate mofetil; Tac—tacrolimus; CYA—cyclosporin. * Second HTX, initial HTX at age 1.

## Data Availability

The original contributions presented in the study are included in the article/Supplementary Material; further inquiries can be directed to the corresponding author.

## Supplementary Materials

The following supporting information can be downloaded at: https://www.mdpi.com/article/10.3390/ijms25147819/s1.
