# Supplementary material for: Undetected Neuromuscular Disease in Patients after Heart Transplantation
Source: Int J Mol Sci. 2024 Jul 17;25(14):7819. doi: 10.3390/ijms25147819 (PMC11277526; doi:10.3390/ijms25147819)
Supplement: Supplementary file 1 [file ijms-25-07819-s001.zip › ijms-3073856-supplementary.pdf]

Table S1. VUS in patients

	Gene OMIM®	Transcript	Variant	Allele frequency % (gnomAD)	In-silico prediction			ClinVar	Detection in analysis stage
					SIFT	Mutation Taster	Polyphen-2		
Patient #1	*ANKRD1* * 609599	NM_014391.2	c.347C>T, p.(Thr116Met)	0.015	deleterious	benign	possibly damaging	uncertain significance likely benign	cardiomyopathy ROI
	FANCM * 609644	NM_020937.2	c.1663G>A, p.(Val555Ile)-	-	deleterious	disease causing	probably damaging	NA	HP:0001638
Patient #2	*KIF1B* *605995	NM_001365951.3	c.2675+3A>G p.(Pro23661Leu)	0.0044	NA	NA	NA	uncertain significance	HP:0009127
	FBN2 *612570	NM_001999.4	c.7561T>A, p.(Tyr2521Asn)	NA	deleterious	deleterious	probably damaging	NA	HP:0009127
	TNNI3 *191044	NM_000363.4	c.278T>C, p.(Leu93Pro)	0.0008	deleterious	deleterious	probably damaging	uncertain significance	cardiomyopathy ROI
	DNA2 *601810	NM_001080449.3	c.2093G>A	0.003	tolerated	NA	probably damaging	uncertain significance	HP:0009127
	MAPT *157140	NM_001377265.1	c.458A>G	-	tolerated	benign	NA	NA	HP:0009127
	HADHA *600890	NM_000182.5	c.1649G>A, p.(Cys550Tyr)	-	deleterious	deleterious	probably damaging	NA	HP:0001638, HP:0009127
Patient #3	*RYR3* *609599	NM_001036.4	c.3389G>A, p.(Arg1130His)	0.008	tolerated	NA	possibly damaging	-	myopathy ROI
	POMK *615247	NM_032237.3	c.271G>A, p.(Ala91Thr)	0.0032	deleterious	disease causing	possibly damaging	uncertain	myopathy ROI
	LGI4 * 608303	NM_139284.2	c.1286T>C, p.(Ile429Thr)	0.12	deleterious	disease causing	probably damaging	likely benign	HP:0009127
	GATA5 * 611496	NM_080473.4	c.56C>G, p.(Ser19Trp)	0.34	deleterious	disease causing	probably damaging	uncertain	HP:0001638
Patient #4	*RAI1* *607642	NM_030665.4	c.3358G>A p.(Asp1120Asn)	0.0024	tolerated	benign	benign	NA	HP:0009127

HP – human phenotype; ROI – region of interest; NA – not available; del – deleterious.

Table S2. Cardiomyopathy-associated genes and transcripts for ROI search

Gene	Transcript
ABCC9	NM_005691.3
	NM_020297.3
ACTC1	NM_005159.4
ACTN2	NM_001103.3
AKAP9	NM_005751.4
ANK2	NM_001148.5
ANKRD1	NM_014391.2
BAG3	NM_004281.3
CACNA1C	NM_000719.6
	NM_199460.3
CACNA2D1	NM_000722.3
CACNB2	NM_201590.2
	NM_201596.2
CALM1	NM_006888.4
CALM2	NM_001743.6
CALM3	NM_005184.4
CALR3	NM_145046.4
CASQ2	NM_001232.3
CAV3	NM_033337.2
CDH2	NM_001792.4
CRYAB	NM_001885.2
CSRP3	NM_003476.4
CTNNA3	NM_013266.3
DES	NM_001927.3
DMD	NM_004006.2
DPP6	NM_001936.4
	NM_130797.3
DSC2	NM_024422.4
	NM_004949.3
DSG2	NM_001943.4
DSP	NM_004415.3
DTNA	NM_001390.4
EMD	NM_000117.2
EYA4	NM_004100.4
FHL1	NM_001449.4
	NM_001159702.2
FKTN	NM_001079802.1
FLNC	NM_001458.4
GATAD1	NM_021167.4
GLA	NM_000169.2
GNAI2	NM_002070.3
GPD1L	NM_015141.3
HCN4	NM_005477.2
JPH2	NM_020433.4

Gene	Transcript
JUP	NM_002230.3
KCND3	NM_004980.4
KCNE1	NM_000219.5
KCNE2	NM_172201.1
KCNE3	NM_005472.4
KCNH2	NM_000238.3
KCNJ2	NM_000891.2
KCNJ5	NM_000890.4
KCNJ8	NM_004982.3
KCNQ1	NM_000218.2
LAMA4	NM_002290.4
	NM_001105206.2
LAMP2	NM_002294.2
	NM_013995.2
	NM_001122606.1
LDB3	NM_007078.2
	NM_001080116.1
	NM_001080114.1
LMNA	NM_170707.3
MYBPC3	NM_000256.3
MYH6	NM_002471.3
MYH7	NM_000257.3
MYL2	NM_000432.3
MYL3	NM_000258.2
MYLK2	NM_033118.3
MYOZ2	NM_016599.4
MYPN	NM_032578.3
NEBL	NM_006393.2
NEXN	NM_144573.3
PDLIM3	NM_014476.5
PKP2	NM_004572.3
PLN	NM_002667.4
PRDM16	NM_022114.3
PRKAG2	NM_016203.3
RAF1	NM_002880.3
RBM20	NM_001134363.2
RYR2	NM_001035.2
SCN10A	NM_006514.3
SCN1B	NM_001037.4
	NM_199037.4
SCN3B	NM_018400.3
SCN4B	NM_174934.3
SCN5A	NM_198056.2
SDHA	NM_004168.3

Gene	Transcript
SGCD	NM_000337.5
SNTA1	NM_003098.2
TAZ	NM_000116.4
TCAP	NM_003673.3
TGFB3	NM_003239.4
TMEM43	NM_024334.2
TMPO	NM_003276.2
TNNC1	NM_003280.2
TNNI3	NM_000363.4
TNNT2	NM_001001430.2
	NM_000364.3
TPM1	NM_001018005.1
	NM_001018004.1
TRDN	NM_006073.3
TRPM4	NM_017636.3
TTN	NM_001267550.2
TTR	NM_000371.3
VCL	NM_014000.2

Table S3. Skeletal disease-associated genes and transcripts

Gene	Transcript
ACTA1	NM_001100.3
ACTN2	NM_001103.3
ADSSL1	NM_199165.2
ANO5	NM_213599.2
B3GALNT2	NM_152490.4
B4GAT1	NM_006876.2
BAG3	NM_004281.3
BIN1	NM_139343.2
BVES	NM_007073.4
	NM_001199563.2
CACNA1S	NM_000069.2
CAPN3	NM_000070.2
CASQ1	NM_001231.4
CAV3	NM_033337.2
CAVIN1	NM_012232.5
CCDC78	NM_001031737.2
	NM_001378030.1
CFL2	NM_021914.7
	NM_138638.5
CHKB	NM_005198.4
CNTN1	NM_001843.3
COL12A1	NM_004370.5
COL6A1	NM_001848.2
COL6A2	NM_001849.3
COL6A3	NM_004369.3
CRYAB	NM_001885.2
	NM_001289808.2
DAG1	NM_004393.5
DES	NM_001927.3
DMD	NM_004006.2
DNAJB6	NM_058246.3
DNM2	NM_001005360.2
	NM_001005361.3
DPM1	NM_003859.2
DPM2	NM_003863.3
DPM3	NM_153741.1
DYSF	NM_003494.3
	NM_001130987.2
EMD	NM_000117.2
FHL1	NM_001449.4
	NM_001159702.3
	NM_001159699.2
FKRP	NM_024301.4
FKTN	NM_001079802.1
FLNC	NM_001458.4
FXR1	NM_005087.3
GAA	NM_000152.4
GIPC1	NM_202470.2
	NM_005716.4
GMPPB	NM_013334.3
	NM_021971.4
GNE	NM_001128227.2
	NM_005476.7
GOLGA2	NM_004486.4
GOSR2	NM_004287.4
HACD1	NM_014241.3
HNRNPA1	NM_031157.3
HNRNPA2B1	NM_031243.2
HNRNPDL	NM_031372.3
HRAS	NM_005343.3
HSPB8	NM_014365.2
INPP5K	NM_016532.3
ISCU	NM_213595.3
ISPD	NM_001101426.3
ITGA7	NM_002206.2

Gene	Transcript
KBTBD13	NM_001101362.2
KLHL40	NM_152393.3
KLHL41	NM_006063.2
KLHL9	NM_018847.3
KY	NM_178554.5
LAMA2	NM_000426.3
LAMP2	NM_002294.2
LARGE1	NM_004737.5
LDB3	NM_001080116.1
	NM_007078.3
LIMS2	NM_001136037.2
	NM_001161403.3
LMNA	NM_170707.3
LMOD3	NM_198271.4
LRP12	NM_001135703.2
MAP3K20	NM_016653.2
MATR3	NM_199189.2
	NM_018834.6
MB	NM_005368.3
MEGF10	NM_032446.2
	NM_001256545.2
MPDU1	NM_004870.3
MSTN	NM_005259.2
MSTO1	NM_018116.3
MTM1	NM_000252.2
MYBPC3	NM_000256.3
MYH2	NM_017534.5
MYH7	NM_000257.3
MYL1	NM_079420.3
MYL2	NM_000432.3
MYMK	NM_001080483.2
MYO18B	NM_032608.6
MYOT	NM_006790.2
MYPN	NM_032578.3
NEB	NM_001271208.1
ORAI1	NM_032790.3
PAX7	NM_002584.2
PLEC	NM_000445.4
	NM_001135254.2
POGLUT1	NM_152305.2
POMGNT1	NM_017739.3
POMGNT2	NM_032806.5
POMK	NM_032237.4
POMT1	NM_007171.3
POMT2	NM_013382.5
	NM_001077365.2
POPDC3	NM_022361.5
PYROXD1	NM_024854.4
RXYLT1	NM_014254.2
RYR1	NM_000540.2
RYR3	NM_001036.4
SCN4A	NM_000334.4
SELENON	NM_020451.2
SGCA	NM_000023.3
SGCB	NM_000232.4
SGCD	NM_000337.5
SGCG	NM_000231.2
SMCHD1	NM_015295.2
SPEG	NM_005876.4
SPTBN4	NM_020971.2
SQSTM1	NM_003900.4
STAC3	NM_145064.2
STIM1	NM_003156.3
SVIL	NM_003174.3
SYNE1	NM_033071.3

Gene	Transcript
SYNE2	NM_182914.2
	NM_182961.4
TCAP	NM_003673.3
TIA1	NM_022173.3
TMEM43	NM_024334.2
TNNT1	NM_003283.5
TNPO3	NM_012470.3
TOR1AIP1	NM_001267578.1
TPM2	NM_003289.3
TPM3	NM_152263.3
TRAPPC11	NM_021942.5
TRIM32	NM_012210.3
TRIM54	NM_032546.3
TRIM63	NM_032588.3
	NM_187841.3
TRIP4	NM_016213.4
TTN	NM_133378.4
UNC45B	NM_173167.3
	NM_001267550.2
VCP	NM_007126.4
	NM_001267052.2
VMA21	NM_001017980.3

Table S4. Genes under Cardiomyopathy (HP:0001638)

Gene ID	Gene Symbol
57505	AARS2
368	ABCC6
10060	ABCC9
51099	ABHD5
27034	ACAD8
28976	ACAD9
35	ACADS
37	ACADVL
58	ACTA1
70	ACTC1
88	ACTN2
51816	ADA2
103	ADAR
111	ADCY5
55750	AGK
178	AGL
10555	AGPAT2
191	AHCY
9049	AIP
56052	ALG1
7840	ALMS1
286	ANK1
29123	ANKRD11
203286	ANKS6
203859	ANO5
411	ARSB
55210	ATAD3A
513	ATP5F1D
514	ATP5F1E
84833	ATP5MK
4508	ATP6
523	ATP6V1A
91647	ATPAF2
9531	BAG3
583	BBS2
617	BCS1L
650	BMP2
388962	BOLA3
673	BRAF
672	BRCA1
675	BRCA2
83990	BRIP1
26580	BSCL2
708	C1QBP
857	CAV1
859	CAV3
284119	CAVIN1
64072	CDH23
1028	CDKN1C
1062	CENPE
1120	CHKB
493856	CISD2
1201	CLN3
81570	CLPB
493753	COA5
388753	COA6
84334	COA8
91949	COG7
1294	COL7A1
27235	COQ2
51117	COQ4
4512	COX1
1352	COX10

Gene ID	Gene Symbol
84987	COX14
1355	COX15
4513	COX2
4514	COX3
1340	COX6B1
1349	COX7B
1374	CPT1A
1376	CPT2
1410	CRYAB
8048	CSRP3
728294	D2HGDH
50717	DCAF8
1674	DES
1738	DLD
1756	DMD
1760	DMPK
131118	DNAJC19
22845	DOLK
54344	DPM3
1824	DSC2
1829	DSG2
1832	DSP
1837	DTNA
1892	ECHS1
60528	ELAC2
2006	ELN
2010	EMD
5167	ENPP1
2038	EPB42
57724	EPG5
2065	ERBB3
2068	ERCC2
2071	ERCC3
2072	ERCC4
2074	ERCC6
1161	ERCC8
2070	EYA4
2184	FAH
2175	FANCA
2187	FANCB
2176	FANCC
2177	FANCD2
2178	FANCE
2188	FANCF
2189	FANCG
55215	FANCI
55120	FANCL
57697	FANCM
22868	FASTKD2
26235	FBXL4
2273	FHL1
9896	FIG4
79147	FKRP
2218	FKTN
80308	FLAD1
2318	FLNC
55572	FOXRED1
79068	FTO
2395	FXN
2548	GAA
2563	GABRD
2626	GATA4
140628	GATA5

Gene ID	Gene Symbol
57798	GATAD1
283459	GATC
2632	GBE1
2702	GJA5
2717	GLA
2720	GLB1
29925	GMPPB
10020	GNE
79158	GNPTAB
2799	GNS
2719	GPC3
2239	GPC4
83550	GPR101
2934	GSN
2961	GTF2E2
404672	GTF2H5
84705	GTPBP3
2990	GUSB
2992	GYG1
2997	GYS1
105259599	H19-ICR
3033	HADH
3030	HADHA
3032	HADHB
57817	HAMP
3043	HBB
3052	HCCS
3077	HFE
138050	HGSNAT
148738	HJV
3106	HLA-B
3155	HMGCL
3178	HNRNPA1
3181	HNRNPA2B1
3257	HPS1
3265	HRAS
3028	HSD17B10
3418	IDH2
3423	IDS
3425	IDUA
64135	IFIH1
3481	IGF2
3593	IL12B
3643	INSR
3679	ITGA7
3704	ITPA
57158	JPH2
3728	JUP
284058	KANSL1
23522	KAT6B
390594	KBTBD13
3756	KCNH1
3784	KCNQ1
10984	KCNQ1OT1
10661	KLF1
10324	KLHL41
3845	KRAS
3908	LAMA2
3909	LAMA3
3910	LAMA4
3914	LAMB3
3918	LAMC2
3920	LAMP2

Gene ID	Gene Symbol
11155	LDB3
11019	LIAS
55679	LIMS2
51601	LIPT1
4000	LMNA
8425	LTBP4
8216	LZTR1
10459	MAD2L2
5604	MAP2K1
5605	MAP2K2
51776	MAP3K20
4158	MC2R
4210	MEFV
4221	MEN1
92667	MGME1
57534	MIB1
125988	MICOS13
4285	MIPEP
23417	MLYCD
25974	MMACHC
4312	MMP1
4594	MMUT
136647	MPLKIP
56246	MRAP
11222	MRPL3
65080	MRPL44
63931	MRPS14
56945	MRPS22
123263	MTFMT
25821	MTO1
4607	MYBPC3
4624	MYH6
4625	MYH7
4633	MYL2
4634	MYL3
85366	MYLK2
84700	MYO18B
93649	MYOCD
9499	MYOT
51778	MYOZ2
84665	MYPN
114803	MYSM1
4668	NAGA
4669	NAGLU
55739	NAXD
51594	NBAS
653361	NCF1
4535	ND1
4536	ND2
4538	ND4
4540	ND5
4541	ND6
4694	NDUFA1
4705	NDUFA10
126328	NDUFA11
55967	NDUFA12
51079	NDUFA13
4695	NDUFA2
4697	NDUFA4
4700	NDUFA6
4704	NDUFA9
51103	NDUFAF1
91942	NDUFAF2

Gene ID	Gene Symbol
25915	NDUFAF3
29078	NDUFAF4
79133	NDUFAF5
137682	NDUFAF6
4716	NDUFB10
54539	NDUFB11
4709	NDUFB3
4714	NDUFB8
4715	NDUFB9
4719	NDUFS1
4720	NDUFS2
4722	NDUFS3
4724	NDUFS4
4726	NDUFS6
374291	NDUFS7
4728	NDUFS8
4723	NDUFV1
4729	NDUFV2
4703	NEB
284086	NEK8
4758	NEU1
91624	NEXN
4763	NF1
23530	NNT
4841	NONO
4878	NPPA
4893	NRAS
80224	NUBPL
57122	NUP107
4976	OPA1
79728	PALB2
25973	PARS2
5095	PCCA
5096	PCCB
5156	PDGFRA
5160	PDHA1
100131801	PET100
5189	PEX1
5192	PEX10
8799	PEX11B
5193	PEX12
5194	PEX13
5195	PEX14
9409	PEX16
5824	PEX19
5828	PEX2
55670	PEX26
8504	PEX3
5830	PEX5
5190	PEX6
5191	PEX7
5236	PGM1
5256	PHKA2
5261	PHKG2
5264	PHYH
51604	PIGT
5318	PKP2
5350	PLN
5373	PMM2
57104	PNPLA2
5428	POLG
11232	POLG2
55624	POMGNT1

Gene ID	Gene Symbol
84197	POMK
10585	POMT1
29954	POMT2
27068	PPA2
5468	PPARG
79717	PPCS
5500	PPP1CB
63976	PRDM16
51422	PRKAG2
5663	PSEN1
5664	PSEN2
5781	PTPN11
5836	PYGL
5837	PYGM
55278	QRSL1
25782	RAB3GAP2
5888	RAD51
5889	RAD51C
5894	RAF1
10616	RBCK1
282996	RBM20
473	RERE
55159	RFWD3
6016	RIT1
55005	RMND1
6023	RMRP
246243	RNASEH1
10535	RNASEH2A
79621	RNASEH2B
84153	RNASEH2C
7737	RNF113A
100151683	RNU4ATAC
50484	RRM2B
6261	RYR1
6262	RYR2
25939	SAMHD1
1757	SARDH
6331	SCN5A
9997	SCO2
6389	SDHA
644096	SDHAF1
6390	SDHB
6392	SDHD
57190	SELENON
6442	SGCA
6443	SGCB
6444	SGCD
6448	SGSH
6472	SHMT2
8036	SHOC2
6497	SKI
10560	SLC19A2
80704	SLC19A3
6584	SLC22A5
788	SLC25A20
5250	SLC25A3
291	SLC25A4
81031	SLC2A10
55532	SLC30A10
30061	SLC40A1
6521	SLC4A1
84464	SLX4
8243	SMC1A

Gene ID	Gene Symbol
6654	SOS1
10290	SPEG
6708	SPTA1
6710	SPTB
6770	STAR
51684	SUFU
6834	SURF1
23345	SYNE1
23224	SYNE2
51204	TACO1
6901	TAFAZZIN
128989	TANGO2
202018	TAPT1
6897	TARS1
8557	TCAP
7015	TERT
7036	TFR2
7040	TGFB1
7043	TGFB3
51300	TIMMDC1
7084	TK2
26007	TKFC
84233	TMEM126A
55863	TMEM126B
79188	TMEM43
54968	TMEM70
7134	TNNC1
7137	TNNI3
51086	TNNI3K
7139	TNNT2
7156	TOP3A
7167	TPI1
7168	TPM1
7169	TPM2
7170	TPM3
11277	TREX1
9325	TRIP4
4558	TRNF
4566	TRNK
11277	TREX1
9325	TRIP4
4558	TRNF
4566	TRNK
4567	TRNL1
4570	TRNN
4572	TRNQ
4574	TRNS1
4575	TRNS2
4576	TRNT
51095	TRNT1
4577	TRNV
4578	TRNW
10102	TSFM
7273	TTN
7274	TTPA
7276	TTR
56652	TWNK
10587	TXNRD2
29089	UBE2T
197131	UBR1
7386	UQCRFS1
9101	USP8
8239	USP9X

Gene ID	Gene Symbol
55697	VAC14
7414	VCL
7415	VCP
7428	VHL
23230	VPS13A
65082	VPS33A
10352	WARS2
7466	WFS1
7504	XK
7516	XRCC2
7518	XRCC4
64131	XYLT1
64132	XYLT2
51067	YARS2

Table S5. Abnormality of the musculature of the limbs (HP:0009127)

Gene ID	Gene Symbol	Gene ID	Gene Symbol	Gene ID	Gene Symbol
8086	AAAS	400916	CHCHD10	2067	ERCC1
16	AARS1	1134	CHRNA1	2074	ERCC6
215	ABCD1	1140	CHRNB1	1161	ERCC8
51099	ABHD5	1144	CHRND	11160	ERLIN2
51	ACOX1	1145	CHRNE	2137	EXTL3
58	ACTA1	63924	CIDEC	2138	EYA1
60	ACTB	1180	CLCN1	79152	FA2H
51412	ACTL6B	1213	CLTC	84668	FAM126A
88	ACTN2	22866	CNKSR2	2201	FBN2
51816	ADA2	65260	COA7	81545	FBXO38
103	ADAR	25839	COG4	23291	FBXW11
111	ADCY5	1303	COL12A1	121512	FGD4
9289	ADGRG1	1305	COL13A1	2255	FGF10
122622	ADSS1	1291	COL6A1	2257	FGF12
10939	AFG3L2	1292	COL6A2	2263	FGFR2
375790	AGRN	1293	COL6A3	2261	FGFR3
207	AKT1	8292	COLQ	2273	FHL1
5832	ALDH18A1	1311	COMP	79147	FKRP
440138	ALG11	116228	COX20	2218	FKTN
199857	ALG14	1337	COX6A1	2313	FLI1
85365	ALG2	65250	CPLANE1	2316	FLNA
57679	ALS2	1371	CPOX	2317	FLNB
8092	ALX1	126129	CPT1C	2318	FLNC
257	ALX3	729920	CRPPA	2483	FRG1
270	AMPD1	1410	CRYAB	55691	FRMD4A
272	AMPD3	55790	CSGALNACT1	2395	FXN
55129	ANO10	9820	CUL7	2555	GABRA2
203859	ANO5	26999	CYFIP2	2558	GABRA5
8120	AP3B2	9420	CYP7B1	2561	GABRB2
9907	AP5Z1	1605	DAG1	2566	GABRG2
367	AR	55152	DALRD3	2581	GALC
64801	ARV1	55157	DARS2	8139	GAN
427	ASAH1	1639	DCTN1	2617	GARS1
51062	ATL1	1644	DDC	2623	GATA1
23400	ATP13A2	80821	DDHD1	57704	GBA2
476	ATP1A1	23259	DDHD2	2643	GCH1
4508	ATP6	1674	DES	54332	GDAP1
23545	ATP6V0A2	1716	DGUOK	10220	GDF11
523	ATP6V1A	79947	DHDDS	84340	GFM2
540	ATP7B	1725	DHPS	2673	GFPT1
546	ATRX	8449	DHX16	2705	GJB1
8314	BAP1	1756	DMD	29926	GMPPA
23299	BICD2	1760	DMPK	29925	GMPPB
274	BIN1	23312	DMXL2	2782	GNB1
673	BRAF	1763	DNA2	10020	GNE
2972	BRF1	3300	DNAJB2	2906	GRIN2D
26580	BSCL2	10049	DNAJB6	2917	GRM7
686	BTD	56521	DNAJC12	2992	GYG1
11149	BVES	9829	DNAJC6	3030	HADHA
83636	C19orf12	1759	DNM1	3032	HADHB
773	CACNA1A	10059	DNM1L	3035	HARS1
774	CACNA1B	1785	DNM2	348980	HCN1
779	CACNA1S	285489	DOK7	3094	HINT1
823	CAPN1	1798	DPAGT1	3098	HK1
825	CAPN3	54344	DPM3	3145	HMBS
857	CAV1	25778	DSTYK	3178	HNRNPA1
859	CAV3	1778	DYNC1H1	9987	HNRNPDL
83987	CCDC8	8291	DYSF	3209	HOXA13
899	CCNF	10682	EBP	3315	HSPB1
966	CD59	1917	EEF1A2	8988	HSPB3
23097	CDK19	1959	EGR2	26353	HSPB8
1073	CFL2	23065	EMC1	3329	HSPD1
1103	CHAT	2010	EMD	200205	IBA57

Gene ID	Gene Symbol
3508	IGHMBP2
64423	INF2
3679	ITGA7
284058	KANSL1
3735	KARS1
7994	KAT6A
10300	KATNB1
390594	KBTBD13
3736	KCNA1
3737	KCNA2
3745	KCNB1
3766	KCNJ10
1E+08	KCNJ18
3759	KCNJ2
23028	KDM1A
8242	KDM5C
57498	KIDINS220
547	KIF1A
23095	KIF1B
3798	KIF5A
10324	KLHL41
339855	KY
3897	L1CAM
3908	LAMA2
3913	LAMB2
9215	LARGE1
3930	LBR
11155	LDB3
163175	LGI4
55679	LIMS2
3991	LIPE
4000	LMNA
56203	LMOD3
4010	LMX1B
29967	LRP12
4038	LRP4
90678	LRSAM1
8425	LTBP4
1130	LYST
51776	MAP3K20
6885	MAP3K7
4137	MAPT
4141	MARS1
9782	MATR3
8888	MCM3AP
9927	MFN2
4311	MME
22880	MORC2
4358	MPV17
4359	MPZ
4507	MTAP
4524	MTHFR
64419	MTMR14
55149	MTPAP
91574	MTRFR
4552	MTRR
4547	MTTP
4593	MUSK
4604	MYBPC1
79784	MYH14
4620	MYH2
4625	MYH7
4633	MYL2

Gene ID	Gene Symbol
389827	MYMK
4649	MYO9A
9499	MYOT
84665	MYPN
23310	NCAPD3
10397	NDRG1
4704	NDUFA9
4718	NDUFC2
4703	NEB
25977	NECAP1
4744	NEFH
4747	NEFL
4763	NF1
4771	NF2
55768	NGLY1
123606	NIPA1
4915	NTRK2
23636	NUP62
116150	NUS1
23363	OBSL1
4976	OPA1
84876	ORAI1
8106	PABPN1
25973	PARS2
5075	PAX1
5077	PAX3
5095	PCCA
5096	PCCB
5155	PDGFB
5165	PDK3
5191	PEX7
5264	PHYH
5297	PI4KA
63895	PIEZO2
23556	PIGN
51227	PIGP
5290	PIK3CA
9373	PLAA
5339	PLEC
5346	PLIN1
5351	PLOD1
8985	PLOD3
5354	PLP1
5376	PMP22
11284	PNKP
57104	PNPLA2
10908	PNPLA6
87178	PNPT1
56983	POGLUT1
5428	POLG
11232	POLG2
11128	POLR3A
55624	POMGNT1
84892	POMGNT2
84197	POMK
10585	POMT1
29954	POMT2
64208	POPDC3
5456	POU3F4
5468	PPARG
5498	PPOX
5530	PPP3CA
5621	PRNP

Gene ID	Gene Symbol
5631	PRPS1
57716	PRX
5660	PSAP
5781	PTPN11
29920	PYCR2
79912	PYROXD1
7879	RAB7A
5894	RAF1
10743	RAI1
5913	RAPSN
5917	RARS1
5921	RASA1
9939	RBM8A
65055	REEP1
23221	RHOBTB2
246243	RNASEH1
6135	RPL11
6138	RPL15
6141	RPL18
6154	RPL26
6155	RPL27
11224	RPL35
6165	RPL35A
6125	RPL5
6204	RPS10
6210	RPS15A
6218	RPS17
6223	RPS19
6229	RPS24
6231	RPS26
6232	RPS27
6234	RPS28
6235	RPS29
6201	RPS7
50484	RRM2B
6253	RTN2
6261	RYR1
26278	SACS
57167	SALL4
1757	SARDH
6305	SBF1
81846	SBF2
6328	SCN3A
6329	SCN4A
6334	SCN8A
9997	SCO2
57410	SCYL1
6389	SDHA
644096	SDHAF1
6390	SDHB
6392	SDHD
57190	SELENON
51091	SEPSECS
10801	SEPTIN9
6442	SGCA
6443	SGCB
6444	SGCD
6445	SGCG
79628	SH3TC2
10280	SIGMAR1
9990	SLC12A6
284111	SLC13A5
6572	SLC18A3

Gene ID	Gene Symbol
6506	SLC1A2
6576	SLC25A1
60386	SLC25A19
89874	SLC25A21
291	SLC25A4
10463	SLC30A9
9197	SLC33A1
55343	SLC35C1
91252	SLC39A13
79581	SLC52A2
113278	SLC52A3
60482	SLC5A7
6531	SLC6A3
6598	SMARCB1
6605	SMARCE1
23347	SMCHD1
6606	SMN1
6608	SMO
6616	SNAP25
6652	SORD
23111	SPART
6683	SPAST
166378	SPATA5
10290	SPEG
80208	SPG11
51324	SPG21
6687	SPG7
6697	SPR
8878	SQSTM1
27286	SRPX2
6736	SRY
6786	STIM1
6812	STXBP1
51684	SUFU
374969	SVBP
23345	SYNE1
23224	SYNE2
8831	SYNGAP1
8867	SYNJ1
127833	SYT2
23334	SZT2
80222	TARS2
6905	TBCE
6926	TBX3
6910	TBX5
8557	TCAP
55775	TDP1
7015	TERT
10342	TFG
7072	TIA1
7084	TK2
79188	TMEM43
23534	TNPO3
7169	TPM2
7170	TPM3
22906	TRAK1
60684	TRAPPC11
22954	TRIM32
9325	TRIP4
7227	TRPS1
59341	TRPV4
116461	TSEN15
80746	TSEN2

Gene ID	Gene Symbol
79042	TSEN34
283989	TSEN54
90121	TSR2
7273	TTN
56652	TWNK
1890	TYMP
79876	UBA5
7343	UBTF
9101	USP8
6843	VAMP1
7415	VCP
7428	VHL
203547	VMA21
23230	VPS13A
137492	VPS37A
10352	WARS2
9897	WASHC5
51741	WWOX
8565	YARS1
7532	YWHAG
23503	ZFYVE26
118813	ZFYVE27
